# Spinal infection by *Enterobacter hormaechei:* an unusual cause of spondylodiscitis: a case report

**DOI:** 10.1186/s13256-026-06333-z

**Published:** 2026-07-30

**Authors:** Zehra Leyla Yapalak, Mine Filiz, Gökhan Yüce, Tuğrul Hoşbul

**Affiliations:** 1 Department of Medical Microbiology, University of Health Sciences, Gülhane Training and Research Hospital, Ankara, Türkiye; 2 Clinic of Infectious Diseases and Clinical Microbiology, University of Health Sciences, Gülhane Training and Research Hospital, Ankara, Türkiye; 3 Department of Radiology, University of Health Sciences, Gülhane Training and Research Hospital, Ankara, Türkiye

**Keywords:** *Enterobacter hormaechei*, Spondylodiscitis, Spinal infection, Rare pathogen, Gram-negative bacteria, Case report

## Abstract

**Introduction:**

Spondylodiscitis is an infectious condition involving the intervertebral disc and adjacent vertebral bodies. Although *Staphylococcus aureus* and *Mycobacterium tuberculosis* are the most common pathogens, cases caused by Gram-negative bacilli are relatively rare. *Enterobacter hormaechei* is an exceptionally uncommon etiologic agent in this context.

**Case presentation:**

A 69-year-old Turkish male with type 2 diabetes mellitus and a history of prostate adenocarcinoma in remission, with no active immunosuppression, presented with lower back pain and elevated inflammatory markers. Initially suspected to have metastatic spinal involvement, spinal MRI revealed spondylodiscitis at the T12–L1 level. Microbiological evaluation of the needle biopsy specimen yielded *Enterobacter hormaechei*. Species-level identification was established by culture and MALDI-TOF MS, with off-label syndromic PCR testing (BIOFIRE^®^ Joint Infection Panel) providing supportive concordant results. The patient was successfully managed with surgical intervention and appropriate antimicrobial therapy.

**Discussion:**

Spondylodiscitis due to *Enterobacter hormaechei* remains exceedingly rare. This case highlights the importance of considering atypical Gram-negative organisms in spinal infections and underscores the value of advanced microbiological methods for accurate species-level identification.

## Introduction

Spondylodiscitis is a rare but serious condition caused by hematogenous spread, direct inoculation during spinal procedures, or contiguous infection [1, 2]. While *Staphylococcus aureus* and *Mycobacterium tuberculosis* are common causative agents, Gram-negative bacilli such as *Enterobacter* spp. are rarely reported. *Enterobacter hormaechei*, a member of the *Enterobacter cloacae* complex (ECC), has recently emerged as a nosocomial pathogen associated with bloodstream and device-related infections [2, 3]. Reports of spinal infections caused by ECC remain limited, and species-level identification has become more feasible with the use of advanced microbiological techniques such as MALDI-TOF MS and molecular methods.

## Case report

A 69-year-old Turkish male with diabetes mellitus (DM), chronic obstructive pulmonary disease (COPD), and hypertension had been diagnosed with prostate adenocarcinoma 1.5 years earlier and achieved remission after chemotherapy. The patient had no known familial genetic or chronic diseases. His psychosocial history revealed active tobacco smoking and occasional alcohol consumption, but no illicit drug use. He had prior hospitalizations for urinary tract infections, urosepsis, and pneumonia, all of which resolved with appropriate antimicrobial therapy. His previous prostate cancer treatment resulted in sustained remission with no evidence of recurrence.

The patient presented to the emergency department with severe lower back pain and progressive gait impairment over the preceding two months, without systemic features such as fever, chills, or weight loss. There was no history of tuberculosis or contact. On admission, he was afebrile and hemodynamically stable. Physical examination revealed lumbar spine tenderness and limited motion due to pain. Neurological evaluation showed normal motor and sensory function, preserved reflexes, and no sphincter disturbances. The patient was admitted with a preliminary diagnosis of metastatic prostate cancer or spondylodiscitis.

**Fig. 1:**
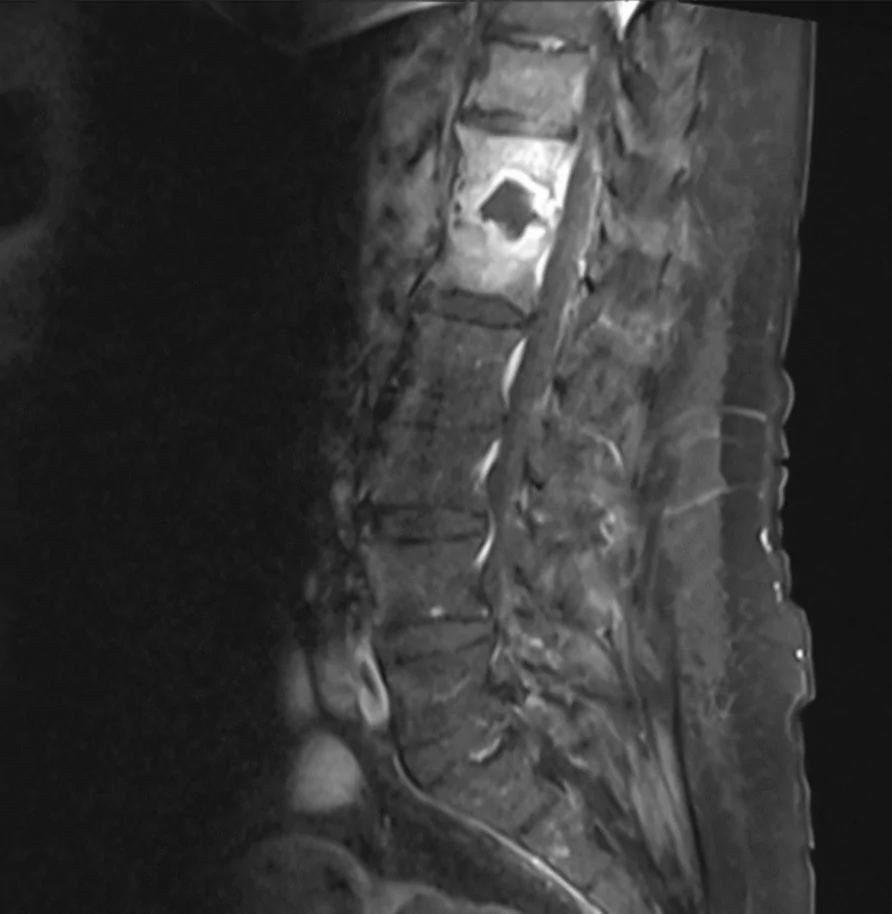
MR image showing a T2-weighted hyperintense and T1-weighted hypointense lesion in the T12–L1 intervertebral disc space, with irregularity and erosion of the adjacent vertebral endplates. At the T10–T11 level, increased signal and mild contrast enhancement are observed on T2-weighted sequences

**Fig. 2:**
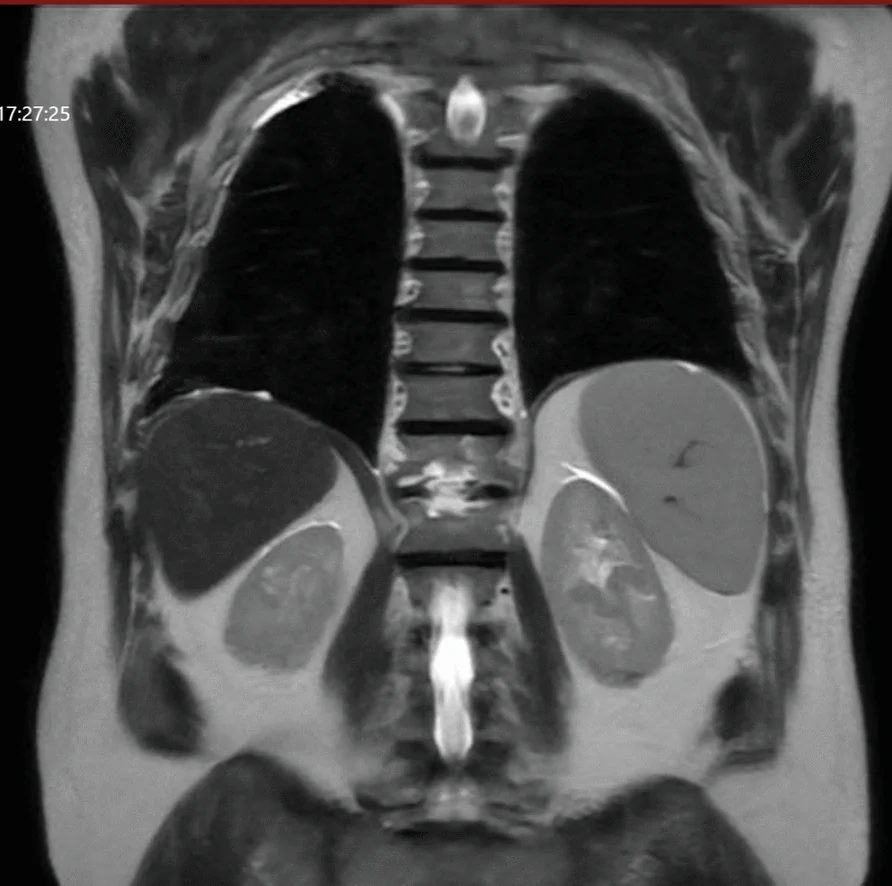
T2-weighted MR images demonstrate hyperintense expansile appearance with centrally necrotic enhancing areas surrounding Schmorl's nodes at the mid-thoracic level

Contrast-enhanced lumbar magnetic resonance imaging (MRI) revealed spondylodiscitis at T12–L1, characterized by disc space narrowing, endplate destruction, and localized enhancement without a frank paravertebral or epidural abscess, alongside suspicious lesions at T10–T11 (Figs. 1 and 2). Gallium-68 prostate-specific membrane antigen positron emission tomography/computed tomography (Ga-68 PSMA PET/CT) showed heterogeneous uptake in both the prostate and T12–L1 vertebrae, initially raising concern for metastasis. However, PSMA uptake is not specific to malignancy and can also occur in infection and inflammation due to PSMA expression in the neovasculature of inflammatory lesions, contributing to the diagnostic challenge in this case.

Laboratory tests showed markedly elevated prostate-specific antigen (PSA) levels (total: 53.8 µg/L, free: 4.82 µg/L). With worsening pain and a C-reactive protein (CRP) level of 155 mg/L, an infectious etiology was suspected. Initial pre-antibiotic blood cultures were obtained at admission, which ultimately remained negative. A computed tomography (CT)-guided biopsy of the lumbar lesion yielded purulent material. Gram staining demonstrated Gram-negative bacilli, and histopathologic examination showed acute suppurative inflammation with neutrophil-predominant infiltrates and focal necrosis, consistent with infectious spondylodiscitis.

Syndromic PCR was used off-label for early guidance and suggested *Enterobacter hormaechei*. Final diagnosis was established by culture, which yielded pure growth of Gram-negative bacilli on blood and eosin methylene blue (EMB) agar, followed by MALDI-TOF MS. The obtained identification score of ≥ 2.0 provides highly reliable species-level identification, which, alongside concordant culture characteristics, validates the *E. hormaechei* diagnosis.

Susceptibility testing (VITEK 2, EUCAST) demonstrated resistance to ceftriaxone, cefepime, and piperacillin–tazobactam, with susceptibility to meropenem (MIC 0.12 mg/L), ciprofloxacin (0.03 mg/L), and trimethoprim–sulfamethoxazole (0.5/9.5 mg/L). All results fell within EUCAST susceptibility breakpoints (meropenem ≤ 0.25 mg/L; ciprofloxacin ≤ 0.25 mg/L; TMP-SMX ≤ 2/38 mg/L for *Enterobacterales*). Intravenous combination therapy with meropenem (1 g q8h) and ciprofloxacin (400 mg q12h) was initiated. This dual regimen was chosen to provide robust empiric coverage given the severity of the spinal infection, to prevent the potential emergence of resistance associated with inducible AmpC β-lactamase in ECC organisms, and to leverage the reliable bone penetration of both agents.

Surgery was indicated because of persistent severe mechanical pain and progressive vertebral body destruction with segmental instability at T12–L1, despite antimicrobial therapy, posing a risk for deformity and neurological compromise. Although MRI also showed signal changes at T10–T11, these were indeterminate and lacked features of active infection; therefore, intervention focused on the symptomatic and unstable T12–L1 segment.

Surgical access was achieved via a standard posterior approach. Following decompression and debridement at the T12-L1 segment, stabilization was performed using posterior pedicle screws. An interbody cage and bone graft were applied to support fusion. Standard intraoperative neuromonitoring was utilized throughout the procedure, and no intraoperative or early postoperative complications were observed. Intraoperative tissue and blood cultures were negative, likely due to prior antibiotic exposure. *E. hormaechei* remained the presumed pathogen given its isolation in pure form from the sterile CT-guided biopsy and the concordant clinical, radiologic, and microbiologic findings.

With clinical improvement and decreasing inflammatory markers, therapy was transitioned on day 18 to oral ciprofloxacin (500 mg BID) plus TMP-SMX (160/800 mg BID) to complete a six-week total course, consistent with vertebral osteomyelitis treatment recommendations.

At six weeks, the patient had complete resolution of back pain and returned to baseline mobility, with normalized inflammatory markers (CRP 155 → 8 mg/L; ESR 72 → 22 mm/h) and no neurological deficits, consistent with clinical cure. Follow-up imaging was not performed, as the patient demonstrated complete clinical and laboratory recovery. This approach is consistent with the IDSA clinical practice guidelines for native vertebral osteomyelitis [4], which suggest that routine follow-up MRI is not necessary in patients with a favorable clinical and laboratory response, as radiologic findings often lag behind clinical improvement. He remained asymptomatic during continued outpatient follow-up. Teaching Point: *Enterobacter hormaechei* should be considered in the differential diagnosis of spondylodiscitis, particularly in patients with a history of recurrent urinary tract infections, underscoring the necessity of advanced molecular diagnostics for accurate identification.

Table 1. Timeline of clinical presentation, diagnostic work-up, treatment, and follow-up. The patient developed progressive lumbar pain two months before admission. CT-guided biopsy confirmed *E. hormaechei* infection. Intravenous meropenem and ciprofloxacin were initiated, followed by surgical stabilization and oral therapy, resulting in complete symptom resolution and normalized inflammatory markers at six weeks.

**Table 1:** Clinical timeline of diagnosis, treatment, and outcome

Time point	Event
2 months before admission	Gradual onset of back pain and difficulty walking
Day 0 (admission)	MRI suspicious; PSMA PET raised concern for metastasis; CRP 155 mg/L
Day 3	CT-guided biopsy → purulent material; Gram-negative bacilli
Day 4	E. hormaechei identified (MALDI-TOF); IV meropenem + ciprofloxacin initiated
Day 18	Surgical stabilization and debridement; switched to oral ciprofloxacin + TMP-SMX
Week 6	Completed antibiotics; full symptom resolution; CRP 8 mg/L, ESR 22 mm/h; no deficits

Time points are presented relative to hospital admission (T = 0)

## Discussion

Pyogenic spondylodiscitis is an uncommon infection involving the nucleus pulposus, often spreading secondarily to the adjacent cartilaginous endplates and vertebral bodies [5, 6]. *S. aureus* is the most frequent causative agent; however, Gram-negative bacilli such as *Enterobacter* species are also occasionally implicated [6]. The genitourinary system is among the most common primary sources, accounting for 17% of cases [2].

A growing incidence of spondylodiscitis has been noted, associated with rising rates of risk factors including diabetes mellitus, chronic kidney disease, intravenous drug use, and immunosuppression [3, 7, 8]. Improved imaging and increased clinical awareness have also contributed to earlier detection [2]. Most patients present with persistent back pain, typically lumbar and exacerbated at night and with movement [1, 3, 8]. Microbiological confirmation is achieved in up to 75% of cases, with an average diagnostic delay of around 30 days [3].

*Enterobacter hormaechei* is a Gram-negative, non-spore-forming bacillus belonging to the ECC and is the most frequently isolated *Enterobacter* species in clinical settings [9]. It is included among the ESKAPE pathogens, known for hospital-acquired infections and resistance mechanisms [10]. First described by O’Hara et al. in 1989, the organism is commonly found in the environment and the human gastrointestinal tract [11]. Its virulence is partly attributed to the chromosomally encoded high-pathogenicity island (HPI) [9, 12]. Healthcare-associated transmission is well recognized, particularly in patients with prior hospitalizations or invasive procedures [12–14]. Given our patient’s previous urinary infections and healthcare exposure, a nosocomial origin was considered plausible. However, pre-antibiotic urine and blood cultures at this admission were negative and no prior *E. hormaechei* isolates were documented, so the precise source remains uncertain. The pathogenesis likely involved hematogenous seeding of the vertebral body, possibly originating from the patient’s history of recurrent urinary tract infections, despite negative cultures at the time of admission. Furthermore, the ability of *Enterobacter* species to form biofilms may have contributed to the persistence of the infection and the necessity for surgical intervention to achieve source control.

Although this pathogen has been isolated from wounds, blood, and respiratory specimens, clinical reports remain limited. Notably, published cases include outbreaks in neonatal intensive care units and sporadic instances of pneumonia, surgical site, or device-associated infections [13–17]. Based on a narrative literature search (PubMed, Google Scholar, and Scopus; January 2000–December 2024; keywords: “*Enterobacter hormaechei*”, “spondylodiscitis”, “spinal infection”), to our knowledge, no prior case with confirmed species-level identification of *E. hormaechei* causing lumbar spondylodiscitis has been reported in a patient without active immunosuppression. Importantly, earlier reports may have been classified simply asECC, as species-level identification has only recently become routine with molecular diagnostics and MALDI-TOF MS [11, 14]. While spondylodiscitis caused by the broader ECC or other Gram-negative bacilli like *Escherichia coli* is well-documented, typically arising from genitourinary sources, species-level confirmed *E. hormaechei* spinal infections remain largely unrecognized. This distinction is critical, as *E. hormaechei* often harbors high-pathogenicity islands and inducible resistance mechanisms that complicate management compared to more common Gram-negative pathogens [9, 12].

In our case, elevated PSA levels and Ga-68 PSMA uptake initially suggested spinal metastasis. It is important to note that PSMA uptake can be seen in infectious and inflammatory conditions, potentially mimicking metastatic disease. However, MRI findings, rising CRP levels, and isolation of the organism from biopsy material confirmed an infectious etiology. Targeted antimicrobial therapy and surgical stabilization resulted in significant clinical improvement. The primary take-home message is that clinicians must maintain a high index of suspicion for atypical Gram-negative pathogens in patients with destructive spinal lesions and a history of recurrent urological infections, even in the absence of severe immunosuppression.

## Conclusion

In conclusion, this case highlights a unique presentation of lumbar spondylodiscitis caused by *E. hormaechei* in a non-immunosuppressed patient. The rarity of this species-level identification underscores the necessity of utilizing advanced proteomic diagnostics, such as MALDI-TOF MS, rather than relying solely on conventional culture. Clinically, physicians should maintain a broad differential diagnosis for spinal lesions mimicking malignancy, particularly in patients with a history of recurrent urological infections. Prompt identification and the implementation of specific combination antimicrobial therapy—leveraging agents with excellent bone penetration and stability against inducible beta-lactamases—coupled with timely surgical debridement, are essential for resolving such complex infections.

## Data Availability

Not applicable.
